# Mature solid teratoma of the uterine cervix: A rare case report and review of the literature

**DOI:** 10.1097/MD.0000000000037451

**Published:** 2024-03-29

**Authors:** Minhua Li, Weiping Zheng

**Affiliations:** a Department of Pathology, Shaoxing People’s Hospital, Shaoxing, Zhejiang, China; b Department of Gynecology, Shaoxing People’s Hospital, Shaoxing, Zhejiang, China.

**Keywords:** cervical polypoid mass, extragonadal teratomas, mature teratomas, uterine cervix

## Abstract

**Rationale:**

Most of the mature teratomas are found in the ovaries. Extragonadal teratomas are extremely rare. To date, there are only a handful of reports of uterine cervical teratomas documented in the English literature.

**Patient concerns:**

Herein we describe a rare case of a 40-year-old patient who was presented to our hospital for a cervical polypoid mass, which was finally confirmed to be mature solid teratoma in uterine cervix.

**Diagnoses:**

Histological examination of the polypoid mass was found to consist of ciliated pseudostratified columnar respiratory epithelium, intestinal epithelium and smooth muscle tissue, adipose tissue and mature glial component, epidermis, and skin adnexa. Meanwhile, no history of abortion, dilatation, and curettage was present in this patient, so implantation of fetal tissue was excluded. Therefore, we make a diagnosis of uterine cervical mature teratoma.

**Interventions:**

Tumorectomy was performed after discovering the cervical polypoid mass.

**Outcomes:**

The patient had been followed-up for next 3 months after surgery and no recurrence was documented until now.

**Lessons:**

Though teratomas of the uterine cervix are extremely rare, more attention should be paid on this rare but possible tumor for appropriate treatment in these patients.

## 1. Introduction

Teratomas are the most frequent germ cell tumors, which mainly develop in midline structures and often occur in the gonads. Most of these tumors are present in children or young adults and usually arise from primordial germ cells. Teratomas could be subcategorized into immature teratoma and mature (solid or cystic) teratoma, which consist of 2 or 3 germ layers, including ectodermal, mesodermal, and endodermal components.

Extragonadal localization in the uterine cervix are extremely rare. The first case of primary mature teratoma in the uterus was described in 1929.^[1]^ However, in the past 3 decades, there were only a handful of reports of uterine cervical mature teratoma cases documented in the English literature to date.^[2–8]^ Of all these uterine cervical teratomas reported, only 3 case reports^[3,5,7]^ were mature cervical teratomas. Our case report was the fourth mature teratomas in the cervix by reviewing the English literature in the past 3 decades. Though, it is suggested that uterine cervical teratomas may be originated from residual fetal tissue.^[8]^ However, there were different views about the possible origin of uterine cervical teratoma as well.^[9]^

In this report, we described a rare case of mature solid teratoma of the uterine cervix in a 40-year-old patient without implantation of fetal tissue, excluding the possibility of origin from residual fetal tissue. Meanwhile, the clinicopathological features of this rare tumor were discussed by reviewing all of the English literature.

## 2. Case presentation

A 40-year-old Chinese female was admitted to our hospital with a chief complaint of finding a uterine cervical polypoid mass for 3 months. Gynecological examination showed a polypoid mass in internal os of uterine cervix measuring about 1.5 cm. Meanwhile, she had a transvaginal ultrasound scan reporting a polypoid mass in uterine cavity with a size of 1.8 × 0.6 cm. Laboratory tests did not show any tumor markers were abnormal. The analysis of blood tests was similar to the normal baseline. Meanwhile, she had no history of abortion, dilatation, and curettage prior to the current presentation.

**Figure 1.**
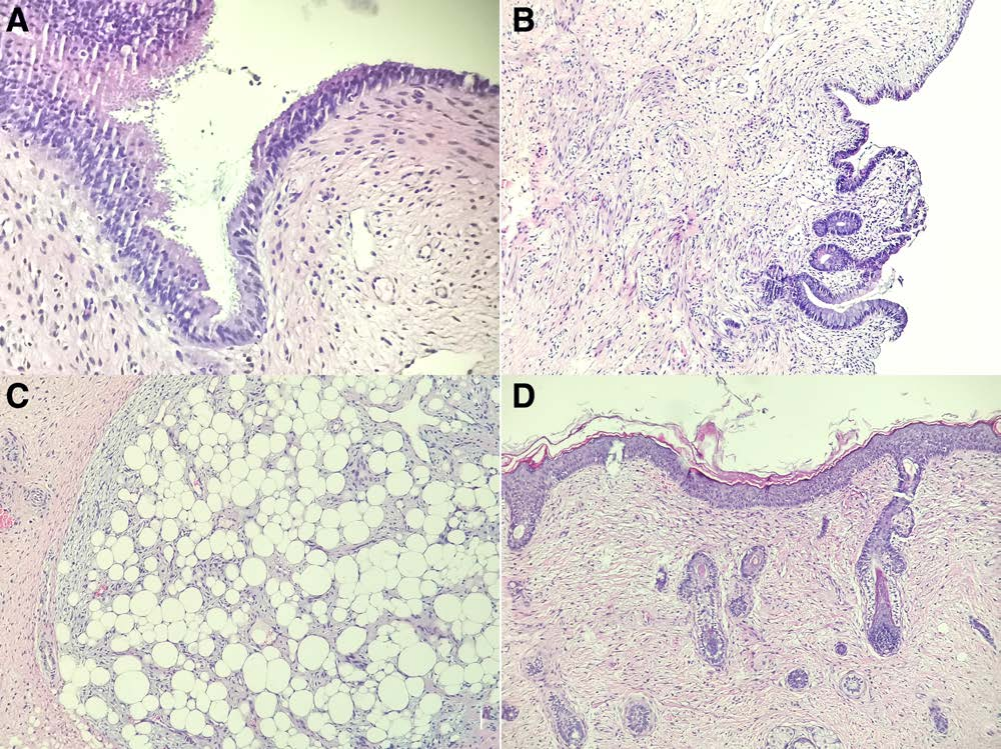
(A) The surface of cervical polypoid mass was covered with ciliated pseudostratified columnar respiratory epithelium (×200). (B) Focal surface of cervical polypoid mass was covered with intestinal epithelium which continued to respiratory epithelium and surrounded by smooth muscle tissue (×100). (C) Adipose tissue mixed with a few mature glial components were showed in the stroma (×100). (D) Epidermis and skin adnexa were obviously revealed in a small polypoid mass (×200).

**Figure 2.**
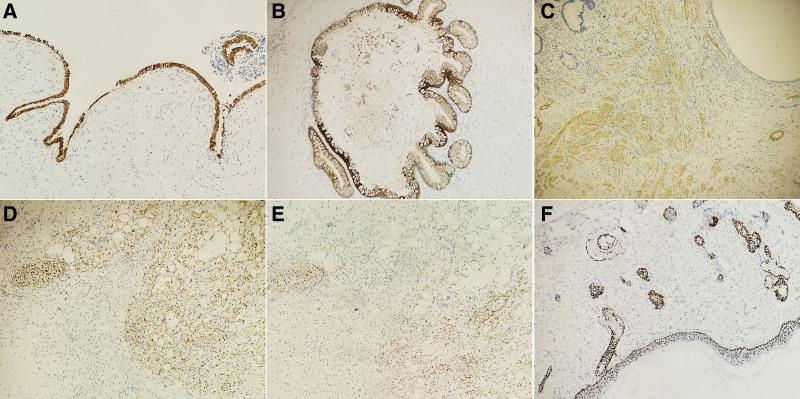
(A) Ciliated pseudostratified columnar respiratory epithelium was positive for CK7 (×200). (B) The positive staining of CK20 was showed in intestinal glands (×200). (C) Smooth muscle tissue was definitely positive for SMA (×100). (D) Strong positive expression of S100 was displayed in adipose tissue and mature glial components (×100). (E) Weak staining of glial fibrillary acidic protein was found in mature glial components (×100). (F) Strong staining of P63 was revealed in the epidermis and skin adnexa in a small polypoid mass (×200). SMA = smooth muscle actin.

Surgically resections of the lesion in uterine cervix and uterine cavity were performed. Macroscopically, multiple solid polypoid masses were showed in uterine cervix, overall measuring 1.5 × 1 cm. Histological examination of the uterine cervical polypoid masses were found to consist of ectodermal, mesodermal, and endodermal components. Most surface of cervical polypoid mass was covered with ciliated pseudostratified columnar respiratory epithelium (Fig. 1A). Focal surface of cervical polypoid mass was covered with intestinal epithelium which continued to respiratory epithelium and surrounded by smooth muscle tissue (Fig. 1B). In the stroma, adipose tissue mixed with a few mature glial components were showed (Fig. 1C), and a few of intestinal glands were surrounded by smooth muscle tissue. The epidermis and skin adnexa was obviously revealed in a small polypoid mass (Fig. 1D). At the same time, when immunohistochemical staining was performed, the results of immunohistochemistry showed that the ciliated pseudostratified columnar respiratory epithelium was positive for CK7 (Fig. 2A), intestinal glands and epithelium were positive for CK20 (Fig. 2B), smooth muscle tissue was positive for smooth muscle actin (Fig. 2C), strong positive expression of S100 was displayed in adipose tissue and mature glial components (Fig. 2D), weak staining of glial fibrillary acidic protein was found in mature glial components as well (Fig. 2E), and positive staining of P63 was showed in epidermis and skin adnexa (Fig. 2F). Moreover, immature components was absent in the whole field. Histological examination of uterine cavity revealed that endometrium was at the stage of proliferative phase without any abnormal presentation. Due to the existence of mature 3 germ layers, the diagnosis of uterine cervical mature teratoma was made.

The patient was disease-free for next 3 months after surgical resection of the tumor and no recurrence was documented until now.

## 3. Discussion

As the most common germ cell tumors, teratomas often occur in the gonads, such as ovary and testicle. Extragonadal germ cell tumors mainly exist in pineal gland, retroperitoneal region, presacral area, and mediastinum. The primary teratomas of uterine cervix are quite rare occurrences.

To our knowledge, there are only a handful of case reports of uterine cervical teratomas that have been described in the English literature in the past 3 decades.^[2–8]^ Of all these uterine cervical teratomas reported, only 3 case reports^[3,5,7]^ were mature cervical teratomas. The first report revealed a mature cervical teratoma with pulmonary differentiation in a 33-year-old women.^[3]^ The second case report described a mature teratoma of the uterine cervix with lymphoid hyperplasia in a 27-year-old woman.^[5]^ The third one reported a case of a 46-year-old patient who presented with multiple solid mature teratomas in the cervix accompanied with immature teratoma in the endometrium and mature teratomas in the ovary.^[7]^ Our case report was the fourth mature teratomas in the cervix by reviewing the English literature in the past 3 decades.

Meanwhile, a rare case of squamous cell carcinoma arising in a teratoma of the uterine cervix was reported in an human immunodeficiency viruses-infected patient.^[4]^ Recently, a rare case report described a unique case of teratocarcinosarcoma in uterine cervix, which was characterized by a mixture of both carcinosarcomatous and teratomatous features, resembling a sinonasal teratocarcinosarcoma.^[10]^ These reports revealed it was possible that the malignant tumors develop from the cervical mature teratomas. Therefore, more attention should be paid on this rare but possible tumor for appropriate diagnosis and treatment in these patients.

There were different views about the possible origin of teratoma. Parthenogenetic origin was the most accepted theory for benign ovarian teratomas. Linder and Power^[11]^ confirmed benign ovarian teratomas arose from parthenogenetically activated ovarian oocytes, which had completed the first meiotic division. They proposed that benign ovary teratomas developed from a single germ cell after the first meiotic division, so benign ovary teratomas were parthenogenetic tumors.^[12]^ As for the possible origins of uterine teratoma, it is suggested that uterine cervical teratomas may be originated from residual fetal tissue.^[8]^ However, a case report proposed that teratomas of the uterus seem to be of a different origin compared with the teratomas of the ovaries.^[9]^ It was possibly that the uterine teratoma was induced by pluripotential stem cell of uterus or primordial germ cell before meiosis I by using short tandem repeats analysis.^[9]^ It was a pity that we did not make short tandem repeats analysis in our case because of the lack of corresponding analysis condition. In our report, no history of abortion, dilatation, and curettage was present in this patient, excluding the possibility of implantation of fetal tissue. Therefore, we support the latter view about the origins of uterine teratoma.

Generally, the primary treatment modalities of uterine cervical teratoma were tumorectomy, conization, or radical hysterectomy with lymphadenectomy.^[6]^ In our case, tumorectomy was performed after discovering the cervical polypoid mass. The patient had been followed-up for next 3 months after surgery and no recurrence was documented until now.

## 4. Conclusions

In conclusion, mature teratomas of the uterine cervix are extremely rare. We present a rare case report of a 40-year-old patient who presented to our hospital for a cervical polypoid mass, which was finally confirmed to be mature solid teratoma in uterine cervix. Although we are not able to provide any evidence for histogenesis of extragonadal teratoma in the uterus cervix, we submitted it to add the records of mature cystic teratomas of the uterine cervix.

## Author contributions

**Investigation:** Minhua Li.

**Project administration:** Weiping Zheng.

**Writing – review & editing:** Weiping Zheng.

**Writing – original draft:** Minhua Li.
